# Supplementary material for: Active strategies for multisensory conflict suppression in the virtual hand illusion
Source: Sci Rep. 2021 Nov 24;11:22844. doi: 10.1038/s41598-021-02200-7 (PMC8613215; doi:10.1038/s41598-021-02200-7)
Supplement: Supplementary file 2 — Supplementary Information 2. [file 41598_2021_2200_MOESM2_ESM.pdf]

Supplementary information

Active Strategies for Multisensory Conflict Suppression in the Virtual Hand Illusion
Lanillos, Pablo[1]*, Franklin, Sae[2], Maselli, Antonella[3], Franklin, David W.[4]

[1]Donders Institute for Brain, Cognition and Behaviour. Artificial intelligence department, Radboud University, the Netherlands.
[2]Institute for Cognitive Systems, Technical University of Munich, Munich, Germany
[3]Institute of Cognitive Sciences and Technology, National Research Council (CNR), Italy
[4]Neuromuscular Diagnostics, Dept. of Sport and Health Sciences,Technical University of Munich, Munich, Germany

* Corresponding author: Pablo Lanillos. Email: p.lanillos@donders.ru.nl

Body-ownership and proprioceptive drift study

We performed a preliminary study to assess the proprioceptive drift and level of body-ownership induced with our experimental setup. Eight participants (a different group from the main study) were tested under the same experimental setup as for the action paradigm. We measured the proprioceptive drift and the body-ownership score. The experiment had ten blocks (five synchronous and five asynchronous), where the virtual hand was placed in one of the three locations: Left, Center, Right. The implementation of the synchronous and asynchronous conditions was the same as in the main experiment.

Equally as the main experiment, participants were seated on the experimental chair, in front of a table, following the instructions of the experimenter. Once the chair was adjusted to the needed height, the experimenter attached the vibrator to the middle point of the hand dorsum. Participants then wore the VR system. The left hand rested on the air sled and the right hand rested on the table. The initial location of the manipulandum was fixed and programmed for all participants. Each condition trial consisted of four phases: 1) *Pre-localization*: Only a table was displayed in the VR and a vertical ruler (depending on the condition) appeared. The participants had to indicate verbally the number on the ruler where they currently perceived the location of the index finger of their left hand. This process was repeated 8 times. As in the main force experiment, between each measurement, participants were asked to look frontal at a cross that appears in the middle of the scene. To avoid the use of the head angle as a cue we injected random noise in the cross location. 2) *Stimulation*: While looking at the cross the virtual arm appeared in the scene in the correct location condition with a bouncing ball on the top of the hand; Participants were instructed to look down towards their index finger. 3) *Post-localization*: Participants were asked to indicate (say the number) where they perceived the index finger of the hand in the same horizontal or vertical ruler as stage 1; Finally, 4) *Resting*: at the end of each trial, participants removed the VR system and rested for 1 minute while filling the illusion questionnaire.

Proprioceptive drift and body-ownership

As expected, proprioceptive drifts were found in the left and right conditions (Figure 1A). Perceptual drifts effects were also found in the asynchronous condition indicating that random vibrations were also integrated although to a lesser degree. The virtual immersion

made that even in the asynchronous condition participants experienced some degree of partial body-ownership, as emerging from by the subjective evaluation of their level of body-ownership towards the virtual hand. Figure 1B shows the questionnaire results using a seven-point Likert scale (3 indicating strong agreement and –3 indicating strong disagreement). Although participants could detect the temporal misalignment of the VT stimulation in the asynchronous condition, as shown by scores to question Q2, this did not completely prevent participants to experience ownership illusion over the virtual hand also in the asynchronous condition (as shown in the positive values of Q1 and Q3).

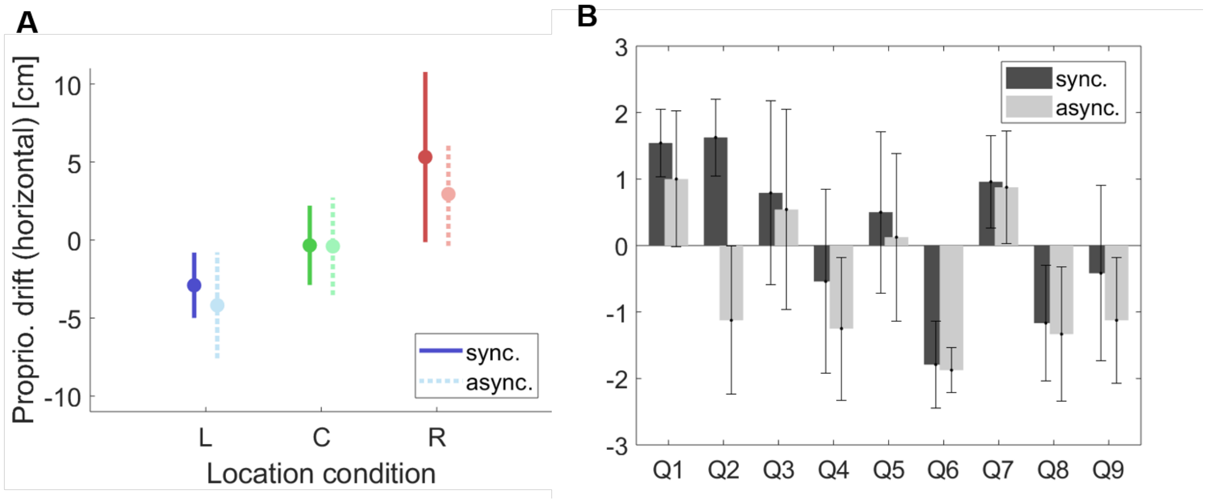

Figure 1: (**A**) Proprioceptive drift for synchronous (continuous line) and asynchronous (dashed line) for the three VR arm location conditions (Left, Center, and Right). (**B**) Body-ownership scoring results from a questionnaire answered by the participants using a 7-point Likert scale (3 indicating strong agreement and -3 indicating strong disagreement).

Questionnaire

The illusion questionnaire was developed by adapting the one presented in Nina et al.[1] to virtual environments with previously developed questionnaires for virtual reality RHI[2,3]. It is compounded by nine questions, where Q1-Q3 describe the RHI effect[4] and Q4-Q9 are control questions.

1. It seemed as if I was feeling the vibration in the location of the virtual arm.
2. Sometimes I had the sensation that the vibration I felt in my hand was caused by the contact of the ball with the virtual hand.
3. There were moments in which I felt that the virtual hand was my own hand
4. There were moments where the touch I was feeling came from somewhere between my own hand and the virtual hand.
5. There were moments in which I felt as if my real hand was becoming virtual
6. It seemed as if I might have more than one left hand
7. The virtual hand started to look like my hand, in terms of shape, skin tone, freckles or some other visual aspects.
8. I felt as if the virtual hand was drifting towards the real hand.
9. I felt as if my real hand was drifting towards the virtual hand.

References

1. Hinz, N.-A., Lanillos, P., Mueller, H. & Cheng, G. Drifting perceptual patterns suggest prediction errors fusion rather than hypothesis selection: replicating the rubber-hand illusion on a robot. in *2018 Joint IEEE 8th International Conference on Development and Learning and Epigenetic Robotics (ICDL-EpiRob)* 125–132 (IEEE, 2018).
2. Ma, K. & Hommel, B. Body-ownership for actively operated non-corporeal objects. *Conscious. Cogn.* **36**, 75–86 (2015).
3. Slater, M., Pérez Marcos, D., Ehrsson, H. & Sanchez-Vives, M. V. Towards a digital body: the virtual arm illusion. *Front. Hum. Neurosci.* **2**, 6 (2008).
4. Botvinick, M. & Cohen, J. Rubber hands ‘feel’ touch that eyes see. *Nature* **391**, 756 (1998).
