# Supplementary material for: Active strategies for multisensory conflict suppression in the virtual hand illusion
Source: Sci Rep. 2021 Nov 24;11:22844. doi: 10.1038/s41598-021-02200-7 (PMC8613215; doi:10.1038/s41598-021-02200-7)
Supplement: Supplementary file 3 — Supplementary Information 3. [file 41598_2021_2200_MOESM3_ESM.html]

RHI\_AI\_vPub


# **The predictive brain in action: Involuntary actions reduce body prediction errors**¶

## RHI computational model based on Active Inference¶

**Author: Pablo Lanillos.**

*Assistant Professor Cognitive AI. Donders Institute for Brain, Cognition and Behaviour.
Department of aThe Netherlands.*
**Email**: p.lanillos@donders.ru.nl

Model based on:

- Oliver, G., Lanillos, P., & Cheng, G. (2019). Active inference body perception and action for humanoid robots. arXiv preprint arXiv:1906.03022.
- Hinz, N. A., Lanillos, P., Mueller, H., & Cheng, G. (2018). Drifting perceptual patterns suggest prediction errors fusion rather than hypothesis selection: replicating the rubber-hand illusion on a robot. IEEE international conference on development and learning and on epigenetic robotics (ICDL-Epirob 2018)
- Friston, K. J., Daunizeau, J., Kilner, J., & Kiebel, S. J. (2010). Action and behavior: a free-energy formulation. Biological cybernetics, 102(3), 227-260.

In [1]:

```
import numpy as np
import matplotlib.pyplot as plt
from numpy.linalg import inv
from scipy.stats import norm
```

### Participants model¶

Version note: there is not subject variability (parameters are fixed)

In [2]:

```
class Participant:
    def __init__(self):
        mu_arm = 44.7208  # From humans data
        std_arm = 2.4807  # From humans data

        # Participants differences have been disabled for code debuging.
        mu_bias_real = 0.
        std_bias_real = 0.
        mu_bias_artificial = -3.9440
        std_bias_artificial = 3.9949

        self.L = (std_arm * np.random.randn() + mu_arm) * 0.01  # arm length (cm)
        self.bias_real = 0.  
        self.bias_artificial = 0.          
        
        # Inverse variances or cues precision
        self.Sigma_x_1 = np.diag(np.exp([1., 1., 1.]))        
        self.Sigma_s_1 = np.diag(np.exp([1., 1., 1., 2.]))

        print('s', self.Sigma_s_1)
        print('x', self.Sigma_x_1)
```

### Experimental setup for each trial¶

In [3]:

```
class Trial:
    def __init__(self, location=0, sync=0):
        # position of the virtual hand
        self.location_conditions = np.array([-15, 0, 15])  # L, C, R
        self.distance_fhand = self.location_conditions[location] * 0.01  # in cm
        self.sync = sync
        # Time step
        self.dt = 0.001
        # Trial time
        self.time = 60  # seconds
        # Number of steps
        self.Nt = np.floor(self.time / self.dt)
        # Visuotactile stimulation init
        self.tinit = 1
        # Visuotactile stimulation ends
        self.tend = 50
        # Total time steps for the AI computation
        self.total_steps = np.arange(self.dt, self.Nt * self.dt, self.dt)
        # Real time steps
        self.t1 = np.arange(round(self.tinit / self.dt), round(self.tend / self.dt))

        # Synchrony modelling as a continuous perturbation DISABLED
        
        # Data logging
        self.data = []  # Store data

    def visualize_experiment(self, human, s_r0, s_v0, s_v_hat, rho, x):
        print('Hands location estimation')
        # visualize experiment
        for l in self.location_conditions:
            plt.plot([l * 0.01, l * 0.01], [0, human.L], 'k--', alpha=0.5)

        plt.plot([0, s_v0], [0, human.L * np.cos(x)], 'mo--')  # real hand
        plt.text(0 + 0.005, human.L - 0.1, 'real hand', color='m')
        plt.plot([s_r0, s_r0], [0, human.L], 'b.-')  # VR hand
        plt.text(s_r0 + 0.005, human.L - 0.13, 'VR hand', color='b')
        plt.plot([s_v_hat], [human.L], 'r.-', markersize=20, alpha=0.5)  # Predicted hand horizontal location
        plt.text(s_v_hat + 0.01, human.L - 0.01, 'predReal', color='r')
        plt.plot([rho], [human.L], 'b.-', markersize=20, alpha=0.5)  # Predicted VR hand horizontal location
        plt.text(rho - 0.04, human.L - 0.04, 'predVR', color='b')

        plt.grid()
        plt.show()

    def reset_data(self):
        self.data = []

    def visualize_data(self):
        w = 8
        h = 4
        data = np.array(self.data).T
        steps = self.total_steps[0:data.shape[1] + 1]

        # arms location
        plt.figure(figsize=(w, h))
        plt.plot(steps, np.ones(len(steps)) * self.distance_fhand, '-.k',
                 label='VR arm location')  # artificial hand location
        plt.plot(steps, data[2, :], '--', color=[0.2, 0.2, 0.5],
                 label=r'$\rho$ - causal variable VR')  # artificial hand location
        plt.plot(steps, data[3, :], linewidth=2, color=[0.8, 0.3, 0.3],
                 label=r'$g(\mu)$ - estimated hand location')  # real hand prediction
        plt.plot(steps, data[17, :], '--', color='k', label='real arm location')  # real hand location (static)
        plt.legend()
        plt.xlabel('time (s)')
        plt.ylabel('horizontal location (cm)')
        plt.ylim([-0.2, 0.2])
        plt.show()

        plt.figure(figsize=(w, h))
        plt.title('Real arm variables')
        plt.plot(steps, data[6, :] * 180. / np.pi, linewidth=2, color='r', label=r'$x$')
        plt.plot(steps, data[7, :] * 180. / np.pi, linewidth=2, color='b', label=r'$x^{\prime}$')
        plt.xlabel('time (s)')
        plt.ylabel('joint angles (deg, deg/s)')
        plt.grid()
        plt.legend()
        plt.show()

        plt.figure(figsize=(w, h))
        plt.title('Inferred Body state (Brain variables)')
        plt.plot(steps, data[0, :] * 180. / np.pi, linewidth=2, color='r', label=r'$\mu$')
        plt.plot(steps, data[1, :] * 180. / np.pi, linewidth=2, color='b', label=r'$\mu\prime$')
        plt.plot(steps, data[16, :] * 180. / np.pi, linewidth=2, color='g', label=r'$\mu^{\prime\prime}$')
        plt.xlabel('time (s)')
        plt.ylabel('inferred joint angles (deg, deg/s)')
        plt.grid()
        plt.legend()
        plt.show()

        plt.figure(figsize=(w, h))
        plt.title('Prediction errors')
        plt.plot(steps, data[8, :], linewidth=2, label=r'$e_{s_p}$')  # es
        plt.plot(steps, data[9, :], linewidth=2, label=r'$e_{s_v}$')  # es
        plt.plot(steps, data[13, :], linewidth=2, label=r'$e_{\rho}$')  # erho
        plt.plot(steps, data[11, :], linewidth=2, label=r'$e_{\mu_0}$')  # ex
        plt.plot(steps, data[12, :], linewidth=2, label=r'$e_{\mu_1}$')  # ex
        plt.xlabel('time (s)')
        plt.ylabel('Prediction errors')
        plt.legend()

        plt.figure(figsize=(w, h))
        plt.title('dot a')
        plt.plot(steps, data[4, :], color=[0.8, 0.3, 0.3], linewidth=2, label=r'$\dot{a}$')
        plt.grid()
        plt.legend()
        plt.show()

        plt.figure(figsize=(w, h))
        plt.title('Force')        
        plt.plot(steps, data[15, :], color=[0.8, 0.3, 0.3], linewidth=2, label='force')
        plt.grid()
        plt.legend()
        plt.show()
```

### Active Inference model and arm model¶

*(1 degree of freedom, elbow rotation)*

In [4]:

```
## Arm equations and generative functions
class Model:
    def __init__(self, human):
        self.beta = 0.0 # strength of the attractor
        self.sync = 0.00001 # prior likelihood of visuotactile synchrony.
        self.a_gain = 0.01
        self.a_saturation = 1.
        self.k = 1 / 4  # elasticity
        self.phi = 2  # viscosity
        self.m = 1  # mass
        self.L = human.L  # arm length
        self.bias_real = human.bias_real  # perceptual bias
        self.alphawrap = -np.pi / 2.0

    def f_real(self, x, rho, a):   
        # Mass-spring-damper
        return np.array([x[1][0], (a - 80*x[0][0] -self.phi*x[1][0])/self.m, 0.]).reshape(3,1)

    def g_real(self, x):
        return self.L * np.cos(x[0][0] + self.alphawrap)

    def T(self, x):  # transformation to joint
        return -self.L * np.sin(x[0][0] + self.alphawrap)

    def A(self, x, rho):  # attractor
        return self.beta * (rho - self.gv(x))

    def gv(self, x):
        return self.L * np.cos(x[0][0] + self.alphawrap)  # + self.bias_real

    def g(self, x, nu):
        return np.array([x[0][0], x[1][0], self.gv(x), nu]).reshape(4, 1)

    # No attractor
    def f(self, x, rho):
        return np.array([x[1][0],
                             (- self.k * x[0][0]) / self.m,  # Only position component
                             0.]).reshape(3, 1)
    def dgv_x(self, x):
        return self.T(x)

    def dg_dx(self, x):
        return np.array([[1., 0., 0.], [0., 1., 0.], [self.dgv_x(x), 0., 0.], [0., 0., 0.]])

    def dg_dnu(self, rho):
        return np.array([0., 0., 1., -1.]).reshape(4, 1)

    # No attractor
    def df_dx(self, x, rho):
        return np.array([[0., 1., 0.],
                         [-self.k / self.m, 0., 0.],
                         [0., 0., 0.]])

    def df_drho(self, x, rho):
        return np.array([0., -self.T(x) * (self.beta / self.m), 0.]).reshape(3, 1)

    def ds_da(self, x, rho):
        return 1 / self.k

    def set_sync(self, sync):
        self.sync = sync
```

### Run experiment¶

Virtual hand location - location\_condition = (0 - Left, 1 - Center, 2 - Right)

In [5]:

```
# Run experiment
print(' > Generating participant')
human = Participant()
# assign model equations
model = Model(human)

location_condition = 2  # location VR arm 0 - Left, 1 - center, 2 - Right
sync_condition = 1  # synchronous disabled
loc = ("left", "center", "right")
print(' > Building trial: loc:', loc[location_condition], ' sync:', sync_condition, 'experiment visualization below')
e = Trial(location_condition, sync_condition)

## Active inference algorithm
# Initialize variables
# Brain variables
x0 = np.array([0, 0, 0]).reshape(3, 1)  # 1st order generalized coordinates
x = x0  # latent space

# Initial observations
s_r0 = e.distance_fhand  # Initial virtual hand visual location (horizontal)
s_v0 = 0.  # Real hand visual location (horizontal)

# No goal
rho0 = 0.
rho = rho0

# perception of the VR arm as a variable
nu0 = s_r0
nu = nu0

# Visual input initialization
s_v = s_r0 # set to VR arm visual input

# Action initialization
a0 = 0.
a = 0.

s_v_hat = model.gv(x0)  # initial predicted hand location

e.reset_data() # logger
# Visualize initial experiment configuration
e.visualize_experiment(human, s_r0, s_v0, s_v_hat, nu, x0[0][0])

print(' > Running simulation: ', e.time, 'seconds')
for i in range(0, int(e.Nt) - 1):
    ## Causality
    model.set_sync(1.)  # Strength of visual input

    ## sensation (observations)
    s = np.array([x0[0][0],  # joint angle
                  x0[1][0],  # joint velocity
                  nu,  # Visual input s_v -> nu
                  s_v]).reshape(4, 1) # virtual hand
    # sensory prediction error (s - g(\mu))
    es = s - model.g(x, nu)
    es[2] *= model.sync # weighted by causality

    # Dynamics
    fx = model.f(x, rho)

    # Dynamics prediction error  (\mu'- f(\mu))
    ex = np.array([x[1][0], x[2][0], 0.]).reshape(3, 1) - fx

    # partial derivatives
    dgx = model.dg_dx(x)
    dgnu = model.dg_dnu(nu)
    dfx = model.df_dx(x, rho)
    dfrho = model.df_drho(x, rho)
    dex_da = model.ds_da(x, rho)

    ## Variational Free-energy minimization (Oliver & Lanillos 2019, Friston 2010)
    # state update
    xdot = np.array([x[1][0], x[2][0], 0.]).reshape(3, 1)
    xdot += np.dot(dgx.transpose(), np.dot(human.Sigma_s_1, es))
    xdot += np.dot(dfx.transpose(), np.dot(human.Sigma_x_1, ex))
    xdot -= np.array([human.Sigma_x_1[1][1] * ex[1][0], human.Sigma_x_1[2][2] * ex[2][0], 0.]).reshape(3, 1)  #

    x = x + e.dt * xdot  # integration

    # causal vars update (virtual hand visual location)
    nudot = -np.dot(dgnu.transpose(), np.dot(human.Sigma_s_1, es))
    nu = nu + e.dt * nudot[0][0]  # integration

    # action update    
    adot = -model.a_gain * dex_da * (human.Sigma_s_1[0][0] * es[0][0])
    a = a + e.dt * adot  # integration
    a = max(-model.a_saturation, min(model.a_saturation, a))  # saturation

    # Precision optimization
    sigma_v_dot = -0.5 * es[2]**2 + 1.0 / human.Sigma_s_1[2][2]
    sigma_p_dot = -0.5 * es[0]**2 + 1.0 / human.Sigma_s_1[0][0]
    human.Sigma_s_1[2][2] = np.max([1., human.Sigma_s_1[2][2] - e.dt*0.2*sigma_v_dot])
    human.Sigma_s_1[0][0] = np.max([0.01, human.Sigma_s_1[0][0] - e.dt*0.2*sigma_p_dot])

    # real change on the arm (real world model)
    x0dot = model.f_real(x0, rho, a)  # update real state
    x0 = x0 + e.dt * x0dot  # integration 

    s_v0 = model.g_real(x0)  # update real hand location
    s_v_hat = model.gv(x)  # new predicted real hand location

    # Store values for visualization
    e.data.append(
        [x[0][0],
         x[1][0],
         nu,
         s_v_hat,
         adot,
         a,
         x0[0][0],  # 6
         x0[1][0],  # 7
         es[0][0],  # 8
         es[1][0],  # 9
         es[2][0],  # 10
         ex[0][0],  # 11
         ex[1][0],  # 12
         0.,  # 13
         s[0][0],  # 14
         a * np.cos(x0[0][0]),  # a/(human.L*np.sin(np.pi/2 -x0[0][0])) , # 15 force
         x[2][0],  # 16 acceleration
         s_v0,  # 17 Real hand location
         human.Sigma_s_1[0][0], # 18 Precision joint
         human.Sigma_s_1[2][2], # 19 Precision visual
         ]
    )
   

# Visualize final configuration and hands location estimation
e.visualize_data()
print('At the end of the stimulation: ', end='')
e.visualize_experiment(human, s_r0, s_v0, s_v_hat, nu, x0[0][0])
```

```
 > Generating participant
s [[2.71828183 0.         0.         0.        ]
 [0.         2.71828183 0.         0.        ]
 [0.         0.         2.71828183 0.        ]
 [0.         0.         0.         7.3890561 ]]
x [[2.71828183 0.         0.        ]
 [0.         2.71828183 0.        ]
 [0.         0.         2.71828183]]
 > Building trial: loc: right  sync: 1 experiment visualization below
Hands location estimation
```

```
 > Running simulation:  60 seconds
```

```
At the end of the stimulation: Hands location estimation
```

In [ ]:

```

```
